# Lanthanide bioadsorption by the extremophile *Exiguobacterium* sp.: utilizing microbial extracellular polysaccharides for high-value element recovery

**DOI:** 10.3389/fmicb.2025.1575677

**Published:** 2025-07-16

**Authors:** Karem Gallardo, Génesis Serrano, Rodrigo Castillo, Sebastián Michea, Julio I. Urzúa, Dayana Arias, Francisco Remonsellez

**Affiliations:** ^1^Instituto de Ciencias Aplicadas, Facultad de Ingeniería, Universidad Autónoma de Chile, Santiago, Chile; ^2^Programa de Doctorado en Ingeniería Sustentable, Facultad de Ingeniería y Ciencias Geológicas, Universidad Católica del Norte, Antofagasta, Chile; ^3^Departamento de Química Inorgánica, Facultad de Química y de Farmacia, Pontificia Universidad Católica de Chile, Santiago, Chile; ^4^Centro de Materiales para la Transición y Sostenibilidad Energética, Comisión Chilena de Energía Nuclear, Santiago, Chile; ^5^Laboratorio de biología molecular y microbiología aplicada, Centro de Investigación en Fisiología y Medicina de Altura (FIMEDALT), Departamento Biomédico, Facultad de Ciencias de la Salud, Universidad de Antofagasta, Antofagasta, Chile; ^6^Departamento de Ingeniería Química y Medio Ambiente, Facultad de Ingeniería, Universidad Católica del Norte, Antofagasta, Chile; ^7^Centro de Investigación Tecnológica del Agua y Sustentabilidad en el Desierto- Ceitsaza, Facultad de Ingeniería, Universidad Católica del Norte, Antofagasta, Chile

**Keywords:** EPS composition, biofilm, rare earth elements, bioadsorption, isotherms, desorption

## Abstract

Rare Earth Elements (REEs) are essential components in modern technologies but are challenging to extract sustainably. With increasing demand and limited supply, alternative recovery methods such as biosorption have gained attention. In particular, biosorption using extracellular polymeric substances (EPS) offers a promising and environmentally friendly approach. This study explores the potential of *Exiguobacterium* sp. SH31, an EPS-producing extremophilic strain, for the biosorption of six REEs (Y, Pr, Nd, Gd, Tb, and Dy) commonly found in spent mobile phones. EPS production and biofilm formation were evaluated in the presence of REEs at concentrations of 0.1 mM and 1 mM, and at pH values of 7, 7.5, and 8. Biosorption capacity was assessed, and characterization was performed using attenuated total reflectance-Fourier transform infrared spectroscopy (ATR-FTIR) and transmission electron microscopy (TEM). EPS were extracted using ultrasound and EDTA-based protocols for compositional analysis. The SH31 strain tolerated up to 1 mM REEs at all tested pH levels with minimal physiological changes. EPS production increased slightly in the presence of metals, with compositional variations dependent on extraction method and pH. Ultrasound-extracted EPS showed higher polysaccharide content at pH 7 and increased nucleic acids at pH 8, while EDTA-extracted EPS had more proteins at pH 7 and nucleic acids at pH 8. Biofilm formation increased in the presence of metals at pH 7 and was overall higher at pH 8, although reduced compared to the control. Adsorption capacity peaked at pH 8, reaching 87–99% for all REEs, and fitted well to the Langmuir isotherm model, indicating monolayer biosorption. Desorption efficiencies ranged from 30 to 90%, depending on the metal, pH, and concentration. ATR-FTIR analysis identified hydroxyl and carbonyl groups as key functional groups involved in metal binding, with notable spectral changes after REE exposure. TEM images revealed cell surface deformation and nanoparticle formation, but no intracellular metal accumulation, confirming that adsorption occurs through EPS-mediated surface binding rather than bioaccumulation. These findings highlight the potential of *Exiguobacterium* sp. SH31 for REE recovery from e-waste leachates, contributing to sustainable electronic waste revalorization strategies.

## 1 Introduction

Currently, Rare Earth Elements (REEs) are considered critical metals due to their indispensable role in modern technologies, in addition to their challenging extraction from natural resources (Jason Burton, 2022). The demand for REEs is so high that there is a need to develop efficient and sustainable methods for their recovery from different sources. Biosorption emerges as a viable alternative, where extracellular polymeric substances (EPS), that exist as a matrix which surrounds the exterior of microbial cells, can form a matrix known as biofilms and are responsible for biofilm structure (Effendi et al., 2023). EPS contains macromolecules constantly produced by microorganisms, such as proteins, polysaccharides, nucleic acids, and lipids. This is the main reason why EPS has many sites for metal adsorption (Costa et al., 2018). The variation in EPS components can be influenced by bacterial species due to the genetic and metabolic properties of different bacteria (Effendi et al., 2023). Moreover, additional factors such as the food substrate can influence the EPS composition. The method of EPS extraction can also determine the EPS composition in pure cultures. EPS are typically produced through the hydrolysis of macromolecules, cellular lysis, or secretion by microbial cells in response to environmental stress (Cheah et al., 2022). Recent studies showed the potential of bacterial EPS as biosorbents for bioremediation of metals, dyes, and for rare earth elements (Ozaki et al., 2004; Gao et al., 2011; Pan et al., 2017; Jena et al., 2021; Cheah et al., 2022; Serrano et al., 2023). The function of EPS is due to heteropolysaccharide, proteins, nucleic acids, and lipid composition of EPS, which comprise primarily of functional groups such as hydroxyl, carboxyl, methylene, aromatic, and amino (Krishnamurthy et al., 2020). At the correct pH, these functional groups can become negatively charged and effectively attract positively charged metal cations through different mechanisms such as complexation, physisorption, ion exchange, precipitation, and chelation (Gupta and Diwan, 2017). The EPS network can be firmly attached to the cell surface as peripheral capsules (tightly bound EPS, TB-EPS), loosely attached to the cell surface (loosely bound EPS, LB-EPS), or released into the surrounding environment as slimes (slime EPS, S-EPS) (Lin et al., 2014).

Exiguobacterium strains have shown to possess characteristics to remove heavy metal and rare earth elements. The mechanisms by which these bacteria remove metals include biosorption, bioaccumulation, bioreduction/biooxidation, and can often occurring simultaneously (Xiao et al., 2024). The efficiency of biosorption by Exiguobacterium strains can be influenced by several factors, including solution pH, initial heavy metal concentration, contact time, and the physiological state of the cells. Biosorption is a key mechanism for the removal of heavy metal cations such as Cd(II), Pb(II), Cu(II), and Zn(II) by this genus (Xiao et al., 2024). *Exiguobacterium* sp. SH31 has shown EPS in the presence of several metals, such as As, La, Eu, Sm and Gd, to form biofilms or to presumably bioadsorb lanthanides (Pavez et al., 2023; Serrano et al., 2023). It has also been reported that functional groups detected by FTIR of Exiguobacterium strains are mainly OH Stretching, NH_2_, CH Stretching, C = O Stretching, Amine groups C-C and P-O-P (Kalpana et al., 2020). In this context, the aim of this research was to use the EPS producer *Exiguobacterium* sp. SH31 to biosorb the most abundant REEs quantified in spent-mobile phones, as Y, Pr, Gd, Dy, Tb and Nd, to identify and characterize changes in the EPS production, composition, biofilm formation, as well as the adsorption capacity and metal desorption at pH 7, 7.5 and 8, and at 0.1 and 1 mM of metal concentration, to finally confirm whether the mechanism of SH31 to remove these six rare earth is by bioadsorption or other.

## 2 Materials and methods

### 2.1 Spent-mobile phone preparation for elemental analysis

The purpose of analyzing e-waste was to identify the more abundant REEs, in order to use these metals for the following experiments. E-waste was kindly donated. Specimens used were spent mobile phones without lithium batteries and plastics manually crushed to sizes of 2 cm2 approximately and mixed thoroughly. Crushed e-wastes were digested using nitric acid (65%), citric acid (99.5%), sulfuric acid (95–97%), chlorohydric acid (37%) and EDTA (99%) at 70°C, during 2 h. After digestion, samples were filtered in 0.45μm pore size filter and stored for inductively coupled plasma mass spectrometer ICP-MS analysis.

### 2.2 Growth curves of microorganism for metal tolerance

Growth kinetics of SH31 strain were done under 0.1- and 1-mM concentrations of REEs in Luria-Bertani (LB) medium at pH 7, 7.5 and 8. The tested metals used in an independent manner were Y, Nd, Pr, Gd, Dy, and Tb. Growth curves were monitored at optical density of 600 nm (OD600) for 48 h at 37°C with continuous orbital agitation (150 rpm), and 1% bacterial inoculum. LB medium contained metal concentration before adding bacterial inoculum. Measurements were recorded every hour using a microplate reader (InfiniteR 200 Pro, Tecan). Control conditions were equally prepared without the addition of any rare earth element. Each assayed condition was performed in three independent experiments with three technical replicates as well.

### 2.3 Identification of presence and quantification of extracellular polymeric substance

The production and quantification of EPS were performed as previously described (Serrano et al., 2023). Briefly, EPS production was assessed by inoculating the SH31 strain on Congo Red Agar (CRA), which contained 10 g/L tryptone, 5 g/L yeast extract, 1% agar, 40 μg/mL Congo red, 20 μg/mL Coomassie brilliant blue, and aliquots of rare-earth elements aqueous solution, independently added at final concentrations of 0.1 mM and 1 mM each. Approximately 50 μL of SH31 grown to the stationary phase was streaked onto the center of the agar plates. Each sample was then incubated at 30°C for 7 days with daily monitoring.

For EPS quantification, 3 mL of the SH31 strain grown for approximately 24 h were centrifuged for 5 min at 9,000 rpm. The supernatant was removed, and the cells were resuspended in 1 mL of culture media containing 40 μg/mL Congo red. The samples were shaken at 250 rpm at room temperature for 90 min, followed by centrifugation for 5 min at 9,000 rpm. The supernatant was then quantified using a UV-Vis spectrophotometer (Shimadzu 1800) at OD490 nm, compared against a calibration curve with Congo red standards ranging from 5 to 100 μg/mL in LB medium to determine the concentration. Three independent replicates were analyzed for EPS quantification (Serrano et al., 2023).

EPS extraction was performed using two previously described methods, EDTA and ultrasound (Yang et al., 2019). EPS was categorized into two types: loosely bound EPS (LB-EPS) and tightly bound EPS (TB-EPS).

In the case of EPS extracted with EDTA, the LB-EPS treatment considered cells to be centrifuged at 5,000 g for 15 min. The supernatant was stored, and the cells were washed twice with 1 mL of 0.9% NaCl for every 5 mL of cells. All supernatants were combined, filtered through a 0.22 μm membrane filter, and stored as “LB-EPS-EDTA.” For TB-EPS treatment, the pellet was re-suspended in 2 mL of 0.9% NaCl and mixed with an equal volume of 2% Na2 EDTA (53.7 mM). The samples were shaken horizontally at 150 rpm for 4 h at 4°C, followed by centrifugation at 5,000 g for 20 min. The cells were washed twice with 1 mL of 0.9% NaCl for every 5 mL of cells. The supernatants were then stored, filtered through a 0.22 μm membrane filter, and stored as “TB-EPS-EDTA” (Yang et al., 2019).

For EPS using ultrasound treatment, bacterial pellets were re-suspended in 0.9% NaCl to a final volume of 7 mL, followed by sonication at 20 kHz for 2 min. The samples were shaken at 150 rpm for 10 min and then sonicated again for an additional 2 min. The suspension was centrifuged at 5,000 g for 15 min and filtered through a 0.22 μm membrane filter. Finally, the samples were stored as “LB-EPS-ultrasound.” The residues from the previous step were re-suspended in 0.9% NaCl to a final volume of 4 mL. This suspension was sonicated for 10 min and centrifuged at 5,000 g for 20 min. The samples were washed twice with 1 mL of 0.9% NaCl and filtered through a 0.22 μm membrane filter. Finally, the samples were stored as “TB-EPS-ultrasound.”

### 2.4 Biofilm formation assay

Biofilm formation was assessed as previous report (Chauhan et al., 2018). Briefly, *Exiguobacterium* sp. SH31 grew during 24 h, then cultures were re-inoculated (1:100 dilution) and seeded in 24-well plates. After 48 h of incubation at 30°C (without agitation), cultures were removed and washed twice with deionized water. For staining, 1 mL of 0.1% crystal violet solution were added per well and incubated at room temperature for 15 min, then rinsed thrice as described previously. Drying plates overnight was done, and biofilms were quantified by adding 1 mL of 30% glacial acetic acid per well. Absorbance was measured at 550 nm having three analytical and independent replicates.

### 2.5 Metal-sorption assays

for metal adsorption, a multielement calibration curve for metal quantification was performed using a Perkin Elmer Optima 7000 DV inductively coupled plasma mass spectrometer (ICP-MS). Metal standard concentrations ranged from 50 to 1,000 μg/L. Three analytical replicates were measured to obtain the standard deviation of the calibration curve. The mass-to-charge ratios (m/z) used for each element were: 89 m/z for Y, 141 m/z for Pr, 144 m/z for Nd, 157 m/z for Gd, 159 m/z for Tb, and 163 m/z for Dy.

Samples for metal adsorption analysis were evaluated in batch mode. For adsorption method, the growth media were inoculated with 10% of *Exiguobacterium* sp. SH31 and allowed to grow for 24 h at 150 rpm and 30°C. Samples were collected when the microorganisms reached the stationary phase. Subsequently, the cultures were centrifuged at 10,000 rpm, and the supernatants were filtered using 0.45 μm mixed cellulose ester (MCE) filters. The cellular pellet was stored for Fourier transform infrared spectroscopy—attenuated total reflectance (ATR-FTIR) characterization. Filtered samples were stored and quantified by ICP-MS in triplicate. The metal removal efficiency (Ad%) and adsorption capacity (qe) at different pH values were calculated as the following equations (Serrano et al., 2023):


(1) A⁢d%⁢[(C⁢i-C⁢e)/C⁢i]⁢x⁢ 100

(2) q⁢e⁢(m⁢g/g)⁢[(C⁢i-C⁢e)/m]⁢x⁢V

Where Ad% is the metal removal efficiency and qe is the adsorption capacity. Ci and Ce are the initial metal and residual concentrations, respectively. V is the volume of the aqueous solution (L) and m is the mass of the cellular pellet used (g).

Considering the adsorption capacity, adsorption isotherms for every rare-earth element were described at pH values of 7, 7.5 and 8. Isotherm models, Freundlich, Langmuir and Temkin, are represented in the following equations, respectively, as shown in Table 1.

**TABLE 1:** Freundlich, Langmuir, and Temkin isotherm equations and its parameters to be determined.

Isotherm models	Equations	Plot	Adsorption parameters to be determined
Freundlich (Eq. 3)	*qe* = *Kf* $\sqrt[{n}]{Ce}$	*Log qe vs log Ce*	*Kf* = exp (*intercept*) *n* = 1/*slope*
Langmuir (Eq. 4)	*qe* = *qmKlCe*/ 1 + *KlCe*	*Ce*/*qe* vs Ce	*qm* = 1/*slope* *Kl* = *slope*/*intercept* *RL* = 1/1 + *KLCo*
Temkin (Eq. 5)	*qe* = *B* ln (*Kt*) + *B*(*lnCe*)	*qe vs ln (Ce)*	*B* = *slope* *Kt* = *intercept*/*slope*

For desorption studies batch mode was evaluated, as well. The pellet cellular was incubated with 10 mL 0.5 M HCl and shaken at 150 rpm during 24 h at room temperature. Equations 6 and 7 were applied to calculate de desorption capacity (qel) and desorption percentage (D%), respectively.


(3) q⁢e⁢l⁢(m⁢g/g)⁢[C⁢f/m]⁢x⁢V

(4) D%⁢[q⁢e⁢l/q⁢e]⁢x⁢ 100

where Cf is the final concentration at the equilibrium, V is the solution volume (L) and m is the adsorbent mass (g).

### 2.6 Bacterial-sorption characterization

Two techniques were used for pellet cellular analysis after metal adsorption, Fourier transform infrared spectroscopy—attenuated total reflectance (ATR-FTIR) and Transmission electron microscopy (TEM).

As described previously (Serrano et al., 2023), to study the surface chemistry as functional group changes of SH31 strain before and after adsorption of rare-earth elements, attenuated total reflection (ATR) was used as a sampling technique alongside traditional infrared spectroscopy Fourier-transform infrared (FTIR). ATR-FTIR analysis (Perkin Elmer Spectrum 100 spectrometer) was used for identifying the functional groups on the bacterial surface. Bare pellet cellular and microorganisms containing rare-earth elements were loaded on the ATR and analyzed directly for their active functional groups in the wavenumber region of 700–4,000 cm^–1^.

SH31 strain cells were analyzed using TEM both before and after REE adsorption. Samples were initially fixed with glutaraldehyde. Ultrathin sections of both blank and metal-loaded samples were then cut using an ultramicrotome and mounted on copper TEM grids. The distribution and morphology of bacteria containing metals, as well as control samples, were characterized. Images were obtained using a Hitachi^®^ HT7700 TEM system operating at an accelerating voltage of 80 kV in high contrast (HC) mode.

## 3 Results

### 3.1 Elemental analysis of spent-mobile phones

the leached E-waste was analyzed by ICP-MS and the results are shown in Supplementary Figure S1. The ICP-MS analysis showed that the six most abundant rare earth elements in a mixture of spent mobile phones are Nd, Pr, Tb, Gd, Y, and Dy. Moreover, additional acids were tested to leach the E-waste in order to determine the maximal yield, where HNO_3_ showed satisfactory results. In this case, most of the rare earth elements were quantified and exhibited higher concentrations compared to other acids such as HCl, citric acid, H_2_SO_4_, and EDTA (data not shown).

### 3.2 Assessing the growth response of *Exiguobacterium* sp. SH31 to various concentrations of rare earth elements at different pH levels

**FIGURE 1:**
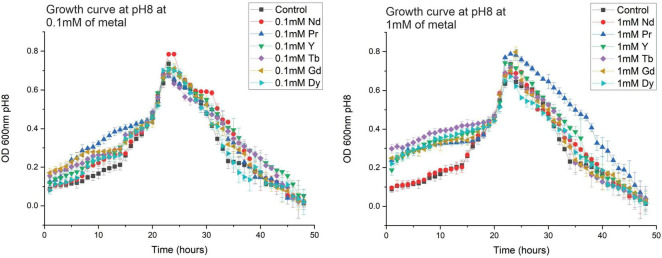
Growth curves of *Exiguobacterium* sp. SH31 at several pH and at 0.1 mM **(A)** and 1 mM **(B)** of Nd, Pr, Y, Tb, Gd and Dy. Error bars represent three independent replicates.

Initially, three different pH values were used to evaluate the tolerance of the SH31 strain to the six most abundant rare earth elements found in electronic waste: Nd^3 +^, Pr^3 +^, Gd^3 +^, Dy^3 +^, Y^3 +^, and Tb^3 +^. The selected pH range was based on the strain’s ability to grow at pH values above 7, while also considering the potential precipitation of rare earth elements at higher pH levels (pH > 8) (Li et al., 2012). It was observed that at higher pH levels, the exponential growth phase started later compared to pH 7. However, no significant differences were noted between the control and the samples containing metals (Figure 1).

### 3.3 Biosorption capacity of *Exiguobacterium* sp. SH31 to Nd^3 +^, Pr^3 +^, Gd^3 +^, Dy^3 +^, Y^3 +^, and Tb^3 +^ and metal desorption

**FIGURE 2:**
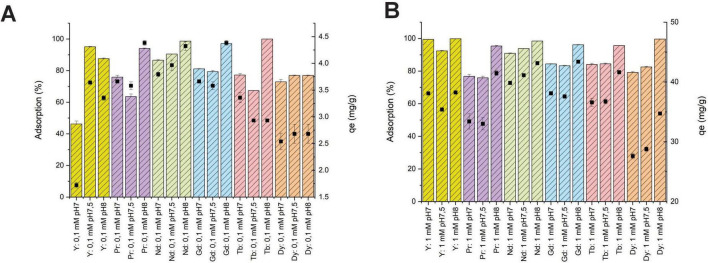
Removal (%) and adsorption capacity (qe) of 0.1 mM **(A)** and 1 mM **(B)** of REEs onto *Exiguobacterium* sp. SH31 at pH7, 7.5 and 8.

determination of removal and sorption capacities of SH31 strain in the stationary phase between pH7 and 8 were performed according to previous report (Serrano et al., 2023). Removal or % sorption of SH31 at pH 7, 7.5 and 8 are shown in Figure 2 and Supplementary Table S1. According to Figure 2 at higher pH the metal adsorption increased (pH8). For instance, bioadsorption at pH8 of 1 mM of Y, Pr, Nd, Gd, Tb, and Dy was more than 95%. In general, adsorption capacity (qe) of REE was high at pH8, for instance, qe of 1 mM of REE at pH8 was more than 35 mg/g (Figure 2 and Supplementary Table S2).

The equilibrium correlations between sorbent and sorbate were evaluated by sorption isotherms according to previous results (Serrano et al., 2023). Experimental isotherms were calculated by the Freundlich, Langmuir, and Temkin isotherm models, and are represented by Equations 3–5, respectively.

The isotherm parameters for the sorption of Y, Pr, Nd, Gd, Tb, and Dy onto the SH31 strain at 30°C were calculated. The highest coefficient value obtained was using Langmuir model, since the R^2^ was 1 (Supplementary Table S3). Moreover, according to the Langmuir model, the separation factor (RL) for each rare-earth element at different pH values, which helps predict the affinity between the sorbent and sorbate, showed an RL between 0 and 1. This indicates that the sorption system is favorable (Supplementary Table S4). Considering the Freundlich and Temkin isotherms, the coefficient values (R^2^) were below 1 for several elements, which immediately disqualifies these models as they do not fit well for this adsorption process (Supplementary Table S3).

Metal desorption performed with 0.5 M HCl showed a higher recovery percentage in samples bioadsorbed at pH 7.5 when initial metal concentration was 0.1 mM. While, at 1 mM the higher performance was achieved in samples adsorbed at pH 8. Ytrium desorption showed the lowest yield, and Nd and Pr showed the best performance in every sample (Supplementary Figure S2).

### 3.4 Extracellular polymeric substances (EPS) production, quantification and composition

**FIGURE 3:**
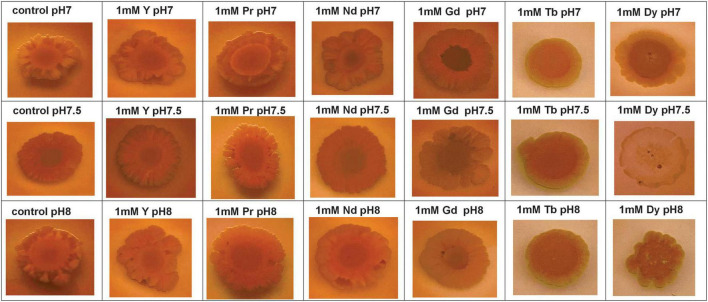
Extracellular polymeric substances production of SH31 strain in absence (control) and in presence of 1 mM of Y^3+^, Pr^3+^, Nd^3+^, Gd^3+^, Tb^3+^, and Dy^3+^ at pH7, 7.5, and 8.

qualitative analysis of EPS production in the SH31 strain was positively observed under all conditions, specifically at 0.1 and 1 mM of REE, using the Congo Red (CR) assay on plates, as shown in Figure 3 and Supplementary Figure S3.

**FIGURE 4:**
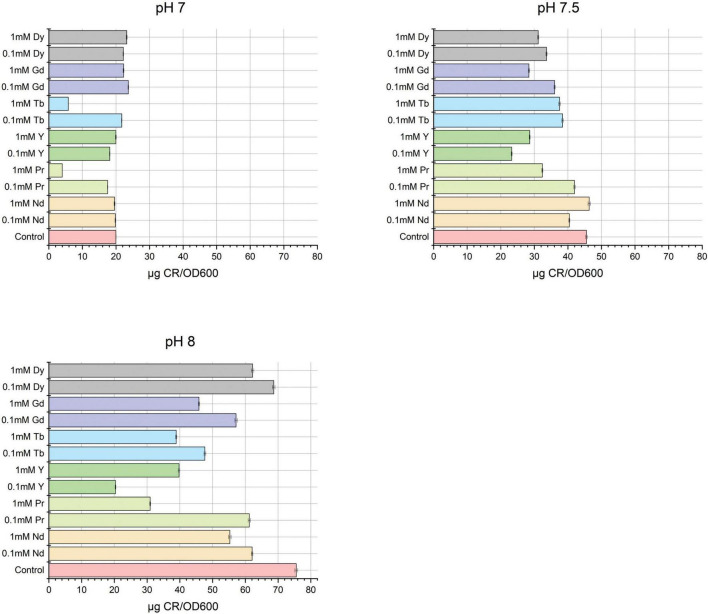
Quantification of total extracellular polymeric substances by Congo red method of SH31 strain after metal contact at pH 7, 7.5, and 8.

Besides the visual changes observed in agar plate assays containing Congo red, quantitative analysis was also performed to confirm changes in the production of extracellular polymeric substances after metal exposure. The CR-binding quantification assay was performed and analyzed using UV-Vis spectroscopy, with results expressed as μg CR/OD600, as previously reported (Serrano et al., 2023). As shown in Figure 4, EPS production increases with pH. However, the total EPS concentration in samples exposed to metal is lower than the control, except for Gd and Dy at pH 7 (Figure 4 and Supplementary Table S5). Moreover, samples containing metal were normalized to the control to determine the fold changes in total EPS due to metal exposure (Supplementary Table S5), and the results showed that most of the total EPS decreased compared to the control at pH 7, 7.5, and 8. However, only Dy^3 +^ and Gd^3 +^ at pH 7 showed a slight increase.

**FIGURE 5:**
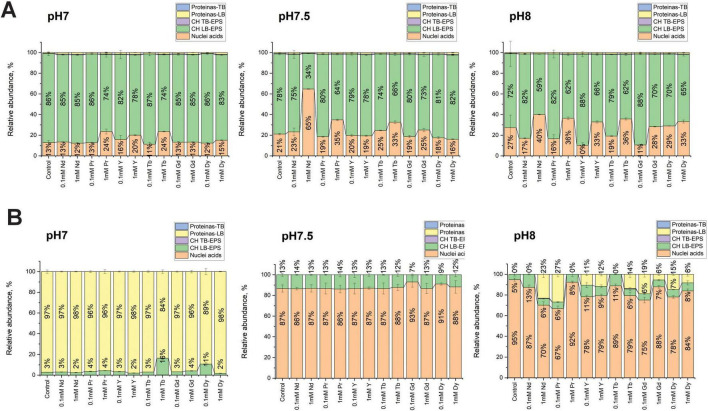
EPS composition of SH31 strain before and after metal exposure, extracted by ultrasound **(A)** and EDTA **(B)** methods. Total nucleic acids, total carbohydrates lightly bound (CH-LB), total carbohydrates tightly bound (CH-TB), total protein lightly bound (LB) and total protein tightly bound (TB) are shown.

Additionally, EPS was extracted using two methods: ultrasound and EDTA, as mentioned previously. Figure 5 presents the quantification of total proteins, carbohydrates, and nucleic acids

Using the ultrasound extraction method, it was observed that carbohydrates were the most abundant component in the LB fraction across all pH conditions, followed by nucleic acids, with total proteins being minimally detected. At pH 7, minor changes were observed in the EPS composition, with a small increase in the nucleic acid content in samples containing 1 mM Pr, Y, and Tb. At pH 7.5, more differences were noted compared to pH 7. For instance, in the presence of 1 mM Nd, the abundance of nucleic acids increased the most, as well as with 1 mM Pr, Tb, and Gd. At pH 8, it was observed that at higher metal concentrations (1 mM), nucleic acids also increased, while at lower metal concentrations (0.1 mM), nucleic acid abundances decreased compared to control samples. Across all pH conditions and metal concentrations, no changes were observed in protein abundances.

Using the EDTA extraction method, more changes were observed in the EPS composition compared to the ultrasound extraction method. At pH 7, proteins were the most abundant component, with a slight increase in carbohydrate abundance in samples containing 1 mM Tb and 0.1 mM Dy. Nucleic acids were poorly quantified in this context. At pH 7.5, a significant increase in nucleic acid abundance was observed, but no major changes across metal concentrations were noted. At pH 8, several changes were observed, such as an increase in protein abundance in samples containing 1 mM Nd, Y, Tb, Gd, Dy, and 0.1 mM Pr, Y, Gd, and Dy. Carbohydrate abundances across all metal concentrations did not show major changes, while nucleic acids, similar to pH 7.5, were the most abundant component in the EPS of the SH31 strain at pH 8

### 3.5 Biofilm formation and quantification

**FIGURE 6:**
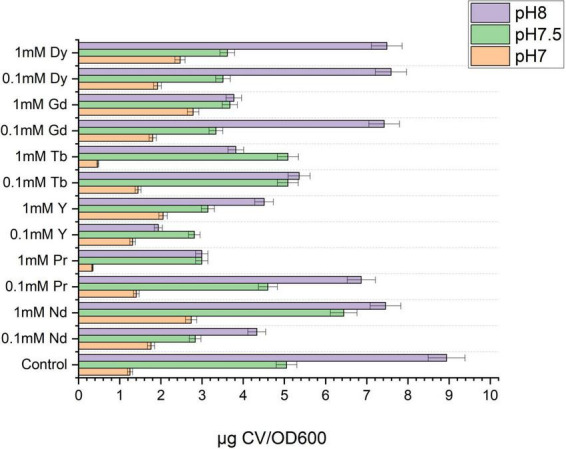
Biofilm quantification by crystal violet assay of SH31 strain at pH7, 7.5 and 8 and at 0.1 and 1 mM of metal concentration.

A qualitative assay was conducted to identify biofilm formation using the crystal violet assay, as shown in Supplementary Figure S4. Initially, positive results were observed at pH 7, 7.5, and 8 compared to the control. However, quantitative results were needed to confirm these findings. Total biofilm production was quantified using crystal violet and UV-Vis spectroscopy. Figure 6 shows the abundances obtained at pH 7, 7.5, and 8 with 0.1- and 1-mM metal concentrations.

The results indicated that, in general, less biofilm is formed at pH 7 than at pH 8. Nevertheless, at pH 7, it was observed that across most metal concentrations, the biofilm formed was higher than the control. As the pH increased, the biofilm produced by the control sample was high, and in the presence of some metals such as Pr, Y, Gd, and Dy, it decreased compared to the control. At pH 8, biofilm production in the control sample was even higher compared to the metal conditions.

### 3.6 Bacterial metal-sorption characterization

**FIGURE 7:**
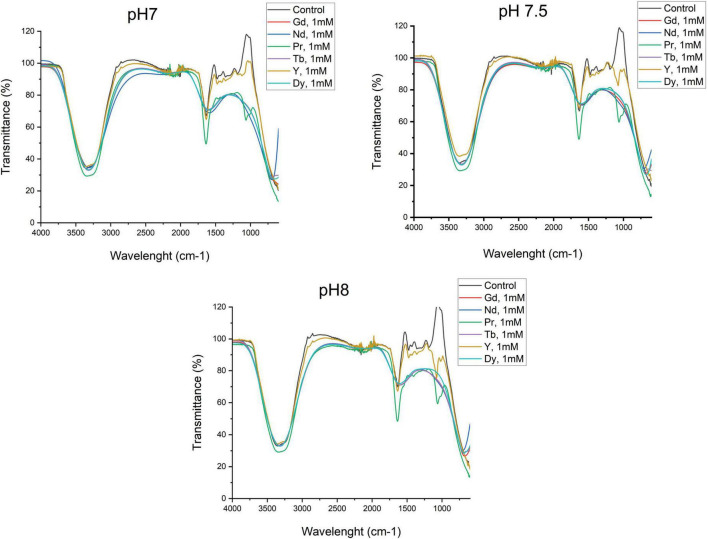
Metal sorption characterization by Fourier transformed infrared spectroscopy of SH31 strain.

Figure 7 shows the FTIR characterization of the SH31 strain before and after metal exposure at pH 7, 7.5, and 8. The FTIR spectra revealed similar patterns between the control, 1 mM Pr, and Y across all pH conditions studied, while the spectra for the other metals also showed similarities. For all samples, both control and metal-containing, characteristic bands of OH functional groups were observed in the region of 3,200–3,500 cm^−1^.

In the control and Y samples, small bands were noted in the 2,800 and 2,900 cm^−1^ regions, corresponding to symmetrical and asymmetrical –CH– vibrations of lipids. Additionally, as illustrated in Figure 7, an intensive absorption band at around 1,638 cm^−1^ and 1,080 cm^−1^ corresponds to Amide I (–CO–) and Amide II (–NH–) in proteins for the control, Y, and Pr samples. A wide and intensive band in the same region, between 1,660 and 1,535 cm^−1^, corresponds to Amide I in proteins for the Gd, Nd, Tb, and Dy samples.

In the 1,250 cm^−1^ region, a band corresponding to phosphate group absorption was detected in the control, Y, and Pr samples, primarily attributed to nucleic acids. Additionally, vibrations of the –COC– group in the cyclic structures of carbohydrates were observed between 1,080 and 1,160 cm^−1^ for the control, Y, and Pr samples. In contrast, this fingerprint region was not well defined in the samples containing Gd, Nd, Tb, and Dy, suggesting that these functional groups may be actively involved in metal interactions.

**FIGURE 8:**
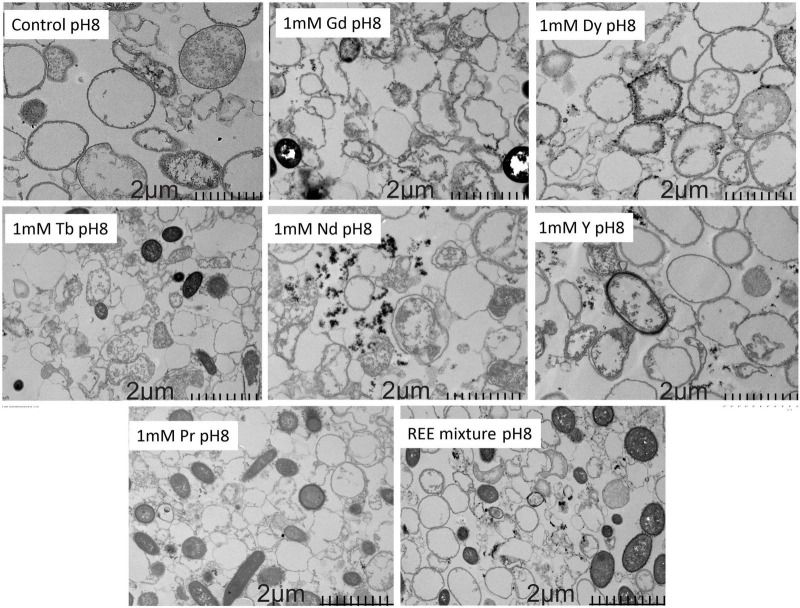
Metal sorption characterization by Transmission electron microscopy.

Transmission electron microscopy analysis was performed to examine the interactions between the SH31 strain and individual metals, as well as a mixture of all six metals, under optimal conditions (pH 8) and at the highest metal concentration. As depicted in Figure 8, the presence of Gd significantly alters cell morphology compared to the control and other metals. For Dy and Y, the metals are clearly adsorbed on the cell surface, whereas Nd is expelled from the cell surface, forming distinct metal agglomerations. In the case of Tb and Pr, the metals completely envelop the cells, likely through interactions with extracellular polymeric substances (EPS). The final image illustrates the SH31 strain’s interaction with all six metals, revealing a combination of behaviors observed individually, such as metal agglomerations, cells coated with metals, metals surrounding the cells, and morphological deformation. These findings indicate that the interactions between the metals and cells are independent of one another and confirm that bioadsorption, rather than bioaccumulation, is the predominant process for Nd, Y, Gd, Dy, Pr, and Tb.

## 4 Discussion

The study presented here investigates the biosorption potential of *Exiguobacterium* sp. SH31 for rare earth elements using extracellular polymeric substances. The findings are significant for advancing sustainable methods for REE recovery, particularly from electronic waste. This discussion will explore the implications of the results, the effectiveness of the biosorption process, and the potential applications and limitations of this approach.

The SH31 strain demonstrated robust tolerance to REEs, maintaining viability at metal concentrations up to 1 mM across all tested pH levels. At 1 mM metal concentration, several changes were observed at pH levels of 7, 7.5, and 8. For instance, at pH 7, the control and metal-containing samples exhibited very similar growth kinetics. At pH 7.5, two groups were observed: the control and Nd-containing samples, which both reached a maximum OD of 0.6, and the second group considered the other elements, which reached an OD of 0.8. At pH 8, the same two groups were observed with similar maximum OD values; however, the lag phase in every condition was longer than at the previous pH levels and metal concentrations. Previous work has shown the growth kinetics of *Exiguobacterium* sp. SH31 in response to La, Eu, Sm, and Gd. The study indicated that no major changes were observed during the exponential phase between the control and metal-containing samples. However, the author noted that the performance of Eu- and Sm-containing cultures was similar during the stationary phase, with a maximum OD of about 0.5, while La- and Gd-containing samples, along with the control, reached an OD of 0.6 (Serrano et al., 2023). An additional report examined the growth kinetics of Exiguobacterium aurantiacum in the presence of NaCl. The analysis indicated that the microorganism could adapt to different salt conditions, achieving good performance after several hours. This information suggests that the presence of NaCl in the growth media might affect the growth kinetics. Therefore, the SH31 strain was grown in LB media without NaCl to avoid any additional effects on bacterial kinetics (Su et al., 2022). Additional studies have shown the growth kinetics of Exiguobacterium strains with different contaminants. For instance, the Exiguobacterium indicum (TBG-PICH-001) grew in the presence of various organic solvents (33% v/v)., such as tetradecane, dodecane, decane, heptane, octane, iso-octane, and hexane (Srivastava et al., 2021). Several differences in growth were observed between tetradecane, dodecane, decane, and control. In contrast, the presence of heptane, octane, and iso-octane reduced the growth kinetics of the microorganism (Srivastava et al., 2021). Moreover, some authors reviewed several Exiguobacterium strains that were used in bioremediation of heavy metals, focusing on the primary mechanism involved in metal removal (Xiao et al., 2024). For instance, E. aurantiacum, *Exiguobacterium* sp. ZM-2, *Exiguobacterium* sp. KCH5, *Exiguobacterium* sp. S8, *Exiguobacterium* sp. Chr-43, *Exiguobacterium* sp. MH3, *Exiguobacterium* sp. CrK19, E.indicum MW1, E.mexicanum CWB-54, *Exiguobacterium* sp. H6, and *Exiguobacterium* sp. PY14 removed Cr(Vl) by bioreduction, *Exiguobacterium* sp. ZM-2, and *Exiguobacterium* sp. E1 removed Cr(Vl) by biosorption, E. profundum PT2 removed As(V) by biosorption, Exiguobacterium sp. ZM-2 removed Cd(II), Ni(II), Cu(II) and Zn(II) by biosorption, *Exiguobacterium* sp. As-9 and E. indicum removed As(V) by bioaccumulation (Xiao et al., 2024).

Initially, removal and biosorption capacity were determined as previous reports (Serrano et al., 2023). The results demonstrated that *Exiguobacterium* sp. SH31 can effectively biosorb REEs, with the best performance observed at pH 8, achieving more than 95% adsorption of 1 mM metal. This high adsorption efficiency is consistent with the Langmuir isotherm model, suggesting monolayer adsorption on a homogeneous surface. Similarly, for qe, more than 35 mg/g was obtained at the same pH and metal concentration. EPS production was slightly increased in the presence of metals, with variations in composition observed depending on the pH and extraction method. It was previously reviewed that several Exiguobacterium strains have been mainly used for heavy metal removal with a good performance (Xiao et al., 2024). For instance, E. aurantiacum removed 80% of Cr(VI) by bioreduction mechanism, *Exiguobacterium* sp. ZM-2 removed 100% of Cr(VI) after 120 min by bioadsorption mechanism with a qe of 29.9 mg/g of living biomass (Alam and Ahmad, 2011; Xiao et al., 2024). An additional strain, as *Exiguobacterium* sp. E1, was also able to remove 83.4% of Cr(VI), *Exiguobacterium* sp. ZM-2 showed an adsorption capacity of 39.9 mg Cd(II), 19.0 mg Ni(II), 20.7 mg Cu (II) and 17.0 mg Zn(II) per gram of non-growing biomass, *Exiguobacterium* sp. H27 remove 56% of 0.1 M Ni(II) and showed a qe of 33 mg/g (Xiao et al., 2024). An additional Exiguobacteriun strain isolated from farmland soil near to Cu, Pb and Zn mine was able to biosorb near to 100% of Cd after 24 h, with a qe of 15.6 mg/g biomass (Park and Chon, 2016). Studies on the biosorption of rare earth elements by Exiguobacterium strains are limited. Recently it was reported the biosorption capacity of the same SH31 strain to four rare earth elements, where the removal of these metals was more than 95% with a maximum adsorption capacity of 23 mg/g.

Acid washing is a common approach for regenerating adsorbents loaded with rare earth elements (REEs) (Yan and Chen, 2024). The higher recovery of metals bioadsorbed at pH 7.5 with an initial concentration of 0.1 mM, using 0.5 M HCl, suggests optimal desorption conditions at this pH. This may result from favorable protonation states of surface functional groups facilitating ion release, as reported previously (Bonificio and Clarke, 2016). Moreover, it was suggested that the preferential binding of lanthanide ions depends on the pH (Bonificio and Clarke, 2016). At this lower concentration, the adsorption sites may be less saturated, allowing for more effective competition between the desorbing HCl and the adsorbed metal ions, leading to higher desorption yields. Additionally, at lower initial concentrations, adsorption sites are less saturated, enhancing competition between desorbing HCl and adsorbed ions. Conversely, at 1 mM concentration, the higher desorption observed at pH 8 indicates shifts in interaction dynamics, such as surface charge changes or altered metal ion speciation, favoring desorption (Bonificio and Clarke, 2016). Differential desorption efficiency among metal ions—yttrium (Y) showing the lowest yield and neodymium (Nd) and praseodymium (Pr) exhibiting the highest—reflects the specificity of the process. This variability likely arises from differences in ionic radii, charge density, and coordination chemistry. For instance, weaker metal-adsorbent complexes in Nd and Pr enhance desorption, while Y’s stronger binding reduces efficiency. Previous studies highlight pH-dependent effects on Y^3 +^ adsorption, with minimal removal at low pH (about 2.4%), which improves as pH increases due to enhanced electrostatic interactions (dos Reis et al., 2024). Despite numerous studies on REE biosorption, metal desorption remains underexplored. Effective recovery rates have been reported in systems such as silica gel coated with polyethyleneimine-immobilized EPS, achieving over 85% recovery and column reuse for up to 10 cycles (Ajao et al., 2020). Mostly, biosorption of rare earth element has been reported, but metal desorption has been poorly identified (Maleke et al., 2019; Costa et al., 2020). These findings underscore the need to optimize both adsorption and desorption strategies, considering pH, concentration, and ion-specific characteristics.

This knowledge gap in desorption processes aligns with the observed adaptive behavior of *Exiguobacterium* sp. SH31, where the good performance is associated with EPS production under different pH and metal conditions. In this context, total EPS quantification was conducted. It was observed that most samples produced slightly less EPS compared to the control at pH 8, unlike at lower pH levels (pH7). In spite of this, the EPS composition changed, it means that total proteins, carbohydrates, and nucleic acid showed differences in their abundances depending on the pH and metal condition. For that reason, two methods were conducted to extract EPS from SH31 strain, ultrasound and EDTA methods. In the first, the EPS extracted with ultrasound exhibited a higher polysaccharide content at pH 7, with minimal changes in EPS composition across all pH levels and metal concentrations compared to the control. While EDTA-extracted EPS showed increased protein levels at pH7, and the nucleic acid content was more pronounced at pH 8 and different metal concentration, suggesting a pH- and metal concentration- dependent modulation of EPS composition. Therefore, the variation in the EPS composition under different conditions suggests that *Exiguobacterium* sp. SH31 can adapt its EPS production in response to metal exposure, optimizing its biosorption capacity. The increase in polysaccharides at pH 7 and nucleic acids at pH 8 indicates a dynamic response to environmental changes, which may enhance the metal-binding sites available for adsorption. Few studies have examined the EPS composition of Exiguobacterium strains. However, recently it was described the EPS composition of SH31 and other strains when they were subjected to arsenic. The results indicated that after 48 h SH31, SH1S21, and SH0S7 secreted higher levels of total EPS (3.3, 2.8, and 2.75 g/L, respectively) compared to control (2.2, 2.6, and 2.5 g/L, respectively), being the SH31 strain the strain that significantly increased EPS production. In addition, the authors indicated that EPS composition showed several differences in the presence of arsenic. Results indicated that high levels of polysaccharides were found in the EPS synthesized by the strains analyzed, representing more than 70% of the total EPS, except for strain SH1S21. Protein and DNA contents were similar in all conditions evaluated, not exceeding 10 and 3%, respectively (Pavez et al., 2023). An additional report also showed that E. aurantiacum EPS was mainly composed of carbohydrates (approximately 66%) when grown in YPMG medium. The study also demonstrated that the medium composition directly affects EPS composition, as the carbohydrate content decreased to 15% when E. aurantiacum was grown in YPM medium. Proteins were scarcely detected in YPMG medium (0.123) and were not detected in YPM medium. Reports on the impact of metals on the EPS composition of various microorganisms are more common. For instance, an additional report indicated that after Cd stress concentration the EPS composition of Pseudomonas aeruginosa changed, the polysaccharides abundance was 26.93% and the protein content was 63.64%; this last one increased the most significantly after Cd exposition, increasing 409.69% when the metal salt corresponds to cadmium nitrate 30. In the case of Alcaligenes faecalis, the same report showed that EPS composition under nonstress conditions was about 7.74% of polysaccharides and 81.14% of proteins. After Cd(NO3)2 stress, Alcaligenes faecalis EPS composition showed a changes in the abundance of polysaccharides and proteins, 6.54% of polysaccharides and 81.95% of proteins. The protein content increased by 52.07% compared to other cadmium salts (Lian et al., 2022). A recent study showed the extracellular polymeric substance impact on contaminated soils and their potential role in heavy metal-bioremediation. The authors indicated that the EPS secreted by *Enterobacter* sp. FM-1 played a vital role in changing soil pH and helped increase soil bio- heavy metal bioavailability. Moreover, the authors showed that secreted EPS by FM-1 strain helped to increase the soil EPS-polysaccharide and EPS-nucleic acid contents; for instance, in highly contaminated soils the LB-EPS addition still increased the EPS-polysaccharide and EPS-nucleic acid contents in the soil by 1.18- and 15.54-fold, respectively. Additionally, EPS of FM-1 strain, specifically LB-EPS and TB-EPS, increased the activities of invertase, urease, alkaline phosphatase, and organic matter in soil, which helped to regulate soil nutrient reserves. Finally, the authors indicated that the addition of different EPS fractions modified the soil microbial community composition to help microbes adapt to a heavy metal contaminated environment (Li et al., 2022). A Bacillus strain showed an increase in total carbohydrates under metal-amended conditions compared to the control condition. The total carbohydrate content in EPS was 2.36, 2.81, 3.41, and 4.38 mg/ml for control, Cd(II), Cu(II), and Pb(II) treated microorganisms, respectively. The total protein content in EPS was measured as 0.103, 0.084, 0.052, and 0.264 mg/ml for control, Cd(II), Cu(II), and Pb(II) treated microorganisms, respectively. Therefore, the author concluded that based on compositional analysis, the proportion of protein content in EPS was lower than that of carbohydrates (Mathivanan et al., 2021).

Moreover, it was observed that SH31 strain can form biofilms under control conditions and in metal contact. It was observed that it increased in the presence of metals at pH 7, which may enhance the stability and effectiveness of the biosorption process. However, at pH 8, although biofilm formation was higher, it was less in the presence of metals compared to the control. This suggests that while biofilms contribute to biosorption, their formation may be inhibited by certain metal concentrations or pH levels. Biofilms can provide a protective environment for microbial cells and increase the surface area available for metal adsorption. A previous study on the same strain showed that biofilm formation was not affected by the presence of the metalloid, which agrees with our results. However, each strain exhibited a different growth pattern. Notably, As(V) induced greater biofilm formation, unlike As(III), which led to a decrease in biofilm formation. These results were confirmed by AFM characterization of biofilm. The authors also indicated that this characteristic is because Exiguobacterium strains have higher expression of genes and proteins related to biofilm formation and response to As stress compared to the untreated strain (Pavez et al., 2023). A quite recent study showed the biofilm formation capacity of *Pseudomonas* chengduensis PPSS-4. Qualitative and quantitative biofilm assays in the presence of multi-metals (Cr, Pb, Cd) at different concentrations of each (25, 50, 100, and 150 mg/L) were done. Biofilm formation by qualitative assay showed a thick ring formation in the air–water interface, which suggested that the microorganism is a strong biofilm former. Biofilm formation in the presence of different concentrations of each metal (25, 50, 100, and 150 mg/L) in multi-metal supplemented media was also observed. The bacterium exhibited weak to strong biofilm formation up to 100 mg/L of each metal. However, there is no biofilm growth at 150 mg/L of the multi-metal treated condition. These qualitative results were confirmed by quantitative biofilm assay, where the isolate showed comparatively higher uptake of Pb(II), Cr(VI), and Cd(II) in biofilm mode as compared to control (Priyadarshanee and Das, 2021). Studies on Bacillus strains have demonstrated their great capacity to form biofilms. The bacterial isolate GH-s29 was shown to form biofilms in the presence of heavy metals; however, no biofilms were observed in the absence of heavy metals. Moreover, the authors indicated that the best biofilm formation was observed for mixed heavy metal culture and As (V), and relatively less biofilm formation was observed in the case of Cr (VI) (Maity et al., 2023). Additional Bacillus strains had been reported to form biofilm against heavy metal stress as well. For instance, *Bacillus* subtilis NCIB 3610 forms a biofilm in the presence of Zn and Cu (Falcón García et al., 2020), and *Bacillus* sp. SFC500-1e was also reported with the ability to form biofilm in the presence of a high concentration of Cr (VI) (Fernandez et al., 2020). The biofilm formations provide a defensive mechanism for the bacterial strains which increases the survivability of bacterial strains.

The functional groups of proteins, carbohydrates, and nucleic acids could be observed when the bacteria produce EPS. In this context, ATR-FTIR results indicated that hydroxyl and carbonyl groups are the predominant functional groups present in samples, observed at 3,373 cm^–1^ and 1,635 cm^–1^, respectively. The observed bands showed wavenumber shifts in the metal presence, and their intensities were also affected. It likely plays a crucial role in the binding of metal ions, as these groups can become negatively charged and attract positively charged metal cations. The main changes were observed in the region below 1,500 cm^–1^ of wavelength, where at pH7.5 and 8, control, Y and Dy samples showed slightly more defined peaks compare to the other metals. These bands correspond to polysaccharides and protein functional groups, as previously reported (Pavez et al., 2023). The peak at the 1,638 cm^–1^ approximately is highly defined in the same samples, control, Y and Dy, while in the other metal conditions it becomes in a wide band. This peak corresponds to carbonyl from amide I. The hydroxy group showed a similar response in the control and metal containing samples, small differences were observed concerning the transmittance abundance only. Similarly, has been shown previously in several studies of the same strain in the adsorption of other lanthanides and arsenic, where the carbonyl and hydroxyl groups are the predominant bands in addition to similar fingerprint areas where the polysaccharides and protein functional groups are shown (Pavez et al., 2023). An additional study done to Pseudomonas aeruginosa and Alcaligenes faecalis showed the FTIR spectra before and after Cd(II) stress/induction. The results exhibited two main bands, which correspond to hydroxyl from polysaccharides and carbonyl groups of amide I. Therefore, the authors inferred that the polysaccharide content of EPS and the increase in C-OH and C = O are related to polysaccharides after metal stress (Lian et al., 2022). Several authors indicated that there is a close relationship between the peak intensity and the concentration of functional groups in the sample (Jiang et al., 2004; Palencia, 2018). Therefore, it can be concluded that in our study the position of the characteristic peaks of the infrared spectrum before and after metal exposure is basically unchanged, which proves that no new functional groups are produced before and after metal adsorption. In the Bacillus strain GH-s29, FTIR spectra formed during different heavy metal treatments showed similar characteristic peaks, such as alcohol (-OH) group at 3,283 cm^–1^, and 1,642 cm^–1^ of secondary amide (CO = NH_2_), among others (Maity et al., 2023). In the case of EPS produced by Pseudomonas aeruginosa OMCS-1 after and before exposure to Cr, Pb and Cd showed a FTIR spectra very similar to SH31 strain. The main bands observed were OH and C = O groups at 3,328 and at 1,634 cm^–1^, respectively. Moreover, the authors showed additional bands observed such as 2,921 cm^–1^ referred to the C–H stretching vibration of –CH_2_ and –CH_3_ groups. The peak at 1,409 cm^–1^ represented the C–O of carboxylate groups or COO– stretches linked with amino acids. The peak at 1,222 cm^–1^ was attributed to N−H stretching of the amide III bond in proteins. The peak at 1,053 cm^–1^ represents the symmetric stretching vibration of P = O of the phosphate group or C−O−C stretching in polysaccharides. The authors validated the involvement of EPS functional groups in heavy metals binding, since after treatment of EPS with Cr(VI), Pb(II), and Cd(II), the position of certain peaks that appeared in the control samples was shifted to varying degrees (Gupta and Diwan, 2017).

The Transmission electron microscopy (TEM) analysis provided significant insights into the interactions between the SH31 strain and various metals under optimal pH conditions (pH 8). The observations highlight diverse morphological changes and metal adsorption behaviors, which are crucial for understanding the bioadsorption mechanisms at play. Morphological changes induced by Gd were particularly notable, as the presence of this metal significantly altered the cell morphology compared to the control and other metals. This suggests that Gd interacts more aggressively or uniquely with cellular structures, potentially affecting cell wall integrity or inducing stress responses; however, no cellular lysis was observed. The lack of lysis suggests that, despite the morphological alterations caused by Gd, the SH31 strain may possess defense mechanisms that mitigate direct toxic effects. This could indicate an adaptive evolutionary capacity of the strain to withstand specific metals, which is crucial for its potential application, as intact bacterial cells exhibit a greater ability to retain metals without releasing toxins into the environment.

The specific morphological changes may be attributed to the ionic properties of Gd, which could disrupt cellular processes or structures. This aligns with a recent study demonstrating that carbon dots synthesized with Gd exhibit an anti-*E. coli* effect, as the metal binds to lipopolysaccharides in the external membranes of E. coli and disrupts their structure (Miao et al., 2025). Additionally, previous studies have also reported changes in bacterial cell morphology upon exposure to metals such as Pb(II), Cr(VI), and Cd(II), with the authors attributing these changes to protective mechanisms, such as those demonstrated by the Acidiphilium symbioticum H8 strain under metal stress (Chakravarty and Banerjee, 2008).

TEM images of the surface adsorption of Dy and Y clearly showed the metal adsorption on the cell surface as dense granules at the nano level (Supplementary Figure S5), indicating a strong affinity between both. This interaction is likely mediated by electrostatic interactions or binding with surface proteins or polysaccharides. The adsorption pattern suggests that these metals do not penetrate the cell membrane but rather form a stable layer on the exterior. Based on this adsorption pattern, it can be inferred that the SH31 strain is particularly effective in the remediation of metals due to its ability to form stable layers on the cell surface without requiring metal internalization. This characteristic not only minimizes potential cytotoxic effects but also simplifies the recovery of adsorbed metals, enhancing their applicability in bioremediation processes. In the case of Neodymium (Nd), it exhibited a distinct behavior by being expelled from the cell surface and forming agglomerations at the nano level (Supplementary Figure S6). This expulsion might be a cellular defense mechanism to prevent metal toxicity. The agglomeration suggests that Nd ions may have a propensity to cluster, possibly due to their chemical properties, which could influence their mobility and bioavailability in environmental contexts. The findings the complete coverage of cells by terbium (Tb) and praseodymium (Pr) indicates a strong interaction with extracellular polymeric substances (EPS). This interaction suggests that EPS plays a critical role in metal binding, possibly through complexation or chelation. Extensive coverage might impact cellular functions by limiting nutrient exchange or inducing mechanical stress. The interaction of SH31 with all six metals simultaneously revealed a combination of the behaviors observed individually. Interestingly, pH appears to influence not only biofilm formation but also the stability of the interaction between metals and bacterial cells. Specifically, the observed changes in biofilm formation at pH 7 and 8 may be related to alterations in the surface charge of the cells, which affect metal attraction. This observation suggests that controlled pH conditions could be a critical factor in optimizing metal adsorption efficiency in bioremediation processes. The presence of agglomerations outside the cells, cell membrane coverage, cell wall interaction, and morphological changes underscores the complexity of multi-metal interactions. The independent behavior of each metal suggests that their binding sites and affinities differ, which might be influenced by their ionic radii, charge, and/or coordination chemistry. In summary, metals observed as dense granules were mainly located on the cell wall after Dy exposure, on the cell membrane after Tb and Pr exposure, on the cell wall and agglomerated outside after Y exposure, on the cell wall and on cell membrane after Gd exposure and agglomerated outside after Nd exposure. Therefore, considering the removal of the six rare earth elements analyzed in this work, it was found that the primary mechanism involved was bioadsorption. Similar results were previously reported, showing that in Pb-uptake electron-dense granules are mainly found on the cell wall and cell membrane. In Cd-uptake electron-dense granules are mainly found on the cell membrane, while in Cu-adsorbed electron-dense granules are mainly found on the outside but located very close to cells (Kim et al., 2007). An older study was done in order to localize the metals several strains. *Pseudomonas* mendocinu, *Arthrobacter* sp., and *Alkaligenes* eutrophus were exposed to silver solutions. The TEM image and EDAX spectra confirmed that metal is located outside the cells and on the cell surface, and in *Alkaligenes* eutrophus is characterized by the production of abundant extracellular polymers (Tsezos et al., 1997). TEM analysis provided us with a comprehensive view of how different metals interact with the SH31 strain. These insights are essential for developing strategies to harness microbial systems for metal recovery and environmental remediation. The findings confirm that bioadsorption is the predominant process rather than bioaccumulation for all metals studied (Nd, Y, Gd, Dy, Pr, and Tb).

This distinction is critical for applications in bioremediation, as bioadsorption involves surface binding without internalization, potentially allowing an easier recovery and reuse of metals. Future studies could explore the molecular mechanisms underlying these interactions to enhance the efficiency and selectivity of bioadsorption processes.

## 5 Conclusion

The study confirms that *Exiguobacterium* sp. SH31 utilizes EPS-mediated bioadsorption to bind rare earth metals, presenting a promising approach for the revalorization of electronic waste. The ability of *Exiguobacterium* sp. SH31 to potentially bioadsorb REEs from spent mobile phones underscores its application in electronic waste recycling. This process offers a sustainable alternative to traditional extraction methods and addresses the growing concern of electronic waste management. Moreover, the specificity of *Exiguobacterium* sp. SH31 for REEs suggests its potential to be tailored for selective recovery of high-demand metals, such as neodymium and terbium, which are critical for advanced technologies. This selective biosorption could offer a competitive advantage over conventional chemical methods, reducing energy consumption and the use of hazardous reagents. The findings also open avenues for integrating microbial biosorption with existing waste treatment processes, potentially creating hybrid systems that combine biological and chemical recovery for enhanced efficiency. Another avenue worth exploring is the valorisation of the recovered REEs for direct reuse in high-tech industries, closing the loop in material cycles. Such advancements would not only mitigate the environmental impact of electronic waste but also contribute to resource independence by reducing reliance on traditional mining of rare earth metals, which often comes with significant ecological and geopolitical challenges.

While the study provides promising results, several limitations must be addressed. The experiments were conducted under controlled laboratory conditions, and scaling up the process for industrial applications may present challenges. Factors such as the presence of competing ions in real-world waste streams, the economic feasibility of the process, and the long-term stability of the biosorption system require further investigation. Future research should focus on optimizing conditions for maximum biosorption efficiency, exploring the genetic and metabolic pathways involved in EPS production, and assessing the environmental impact of large-scale applications. Additionally, investigating the potential for genetic engineering of Exiguobacterium strains to enhance their biosorption capabilities could lead to more efficient recovery methods.

Nevertheless, this study enhances our understanding of *Exiguobacterium* sp. SH31 biosorption mechanisms and contributes to the development of sustainable technologies for critical metal recovery, aligning with global efforts to promote practices of circular economy. The study confirms that *Exiguobacterium* sp. SH31 utilizes EPS-mediated bioadsorption to bind rare earth metals, presenting a promising approach for the revalorization of electronic waste. The ability of *Exiguobacterium* sp. SH31 to potentially bioadsorb REEs from spent mobile phones underscores its application in electronic waste recycling. This process offers a sustainable alternative to traditional extraction methods and addresses the growing concern of electronic waste management. Moreover, the specificity of *Exiguobacterium* sp. SH31 for REEs suggests its potential to be tailored for selective recovery of high-demand metals, such as neodymium and terbium, which are critical for advanced technologies. This selective biosorption could offer a competitive advantage over conventional chemical methods, reducing energy consumption and the use of hazardous reagents. The findings also open avenues for integrating microbial biosorption with existing waste treatment processes, potentially creating hybrid systems that combine biological and chemical recovery for enhanced efficiency.

Another avenue worth exploring is the valorisation of the recovered REEs for direct reuse in high-tech industries, closing the loop in material cycles. Such advancements would not only mitigate the environmental impact of electronic waste but also contribute to resource independence by reducing reliance on traditional mining of rare earth metals, which often comes with significant ecological and geopolitical challenges. While the study provides promising results, several limitations must be addressed. The experiments were conducted under controlled laboratory conditions, and scaling up the process for industrial applications may present challenges. Factors such as the presence of competing ions in real-world waste streams, the economic feasibility of the process, and the long-term stability of the biosorption system require further investigation. Future research should focus on optimizing conditions for maximum biosorption efficiency, exploring the genetic and metabolic pathways involved in EPS production, and assessing the environmental impact of large-scale applications. Additionally, investigating the potential for genetic engineering of Exiguobacterium strains to enhance their biosorption capabilities could lead to more efficient recovery methods. Nevertheless, this study enhances our understanding of *Exiguobacterium* sp. SH31 biosorption mechanisms and contributes to the development of sustainable technologies for critical metal recovery, aligning with global efforts to promote practices of circular economy

## Data Availability

The original contributions presented in the study are included in the article/Supplementary material, further inquiries can be directed to the corresponding authors.
