# Supplementary material for: Lanthanide bioadsorption by the extremophile Exiguobacterium sp.: utilizing microbial extracellular polysaccharides for high-value element recovery
Source: Front Microbiol. 2025 Jul 16;16:1575677. doi: 10.3389/fmicb.2025.1575677 (PMC12307398; doi:10.3389/fmicb.2025.1575677)
Supplement: Supplementary file 1 [file Supplementary_file_1.docx]

Lanthanide Bioadsorption by the extremophile *Exiguobacterium* sp.: Utilizing Microbial Extracellular Polysaccharides for high-value element recovery

Karem Gallardo^1*^, Génesis Serrano^2^, Rodrigo Castillo^3^, Sebastián Michea^1^, Julio I. Urzúa^4^, Dayana Arias^5^, Francisco Remonsellez^6^.

^1^Instituto de Ciencias Aplicadas, Facultad de Ingeniería, Universidad Autónoma de Chile, Santiago, Chile.

^2^Programa de Doctorado en Ingeniería Sustentable, Facultad de Ingeniería y Ciencias Geológicas, Universidad Católica del Norte, Antofagasta, Chile.

^3^Departamento de Química Inorgánica, Facultad de Química y de Farmacia, Pontificia Universidad Católica de Chile, Santiago, Chile.

^4^Centro de Materiales para la Transición y Sostenibilidad Energética, Comisión Chilena de Energía Nuclear, Santiago, Chile

^5^Laboratorio de biología molecular y microbiología aplicada, Centro de Investigación en Fisiología y Medicina de Altura (FIMEDALT), Departamento Biomédico, Facultad de Ciencias de la Salud, Universidad de Antofagasta, Antofagasta, Chile.

^6^Laboratorio de Microbiología Aplicada y Extremófilos, Departamento de Ingeniería Química, Facultad de Ingeniería y Ciencias Geológicas, Universidad Católica del Norte, Antofagasta, Chile.

*** Correspondence:**Karem Gallardo
karem.gallardo@uautonoma.cl

Keywords: EPS composition; Biofilms; Rare Earth elements; Bioadsorption, Desorption, isotherms.

**SUPPLEMENTARY TABLES**

**Table S1: Elemental analysis of E-waste by ICP-MS.**

| REE analyzed | Concentration (ppm) |
| --- | --- |
| [Nd] | 51.200 |
| [Pr] | 19.000 |
| [Tb] | 5.5740 |
| [Gd] | 0.13700 |
| [Y] | 0.40100 |
| [Dy] | 0.14900 |
| [Yb] | 0.07650 |
| [Ce] | 0.03500 |
| [Ho] | 0.00485 |
| [La] | 0.01800 |
| [Eu] | 0.00890 |
| [Sc] | 0.00729 |
| [Sm] | 0.00135 |
| [Tm] | 0.00003 |
| [Lu] | 0.00153 |

**Table S2: Removal or % adsorption of Nd³⁺, Pr³⁺, Gd³⁺, Dy³⁺, Y³⁺, and Tb³⁺ on *Exiguobacterium* sp. SH31, at pH7 (A); pH 7.5(B); pH8 (C)**

| **(A)**  **Samples** | **Average adsorption (%)** | **%RSD** |  | **(B)**  **Samples** | **Average adsorption (%)** | **%RSD** |  | **(C)**  **Samples** | **Average adsorption (%)** | **%RSD** |
| --- | --- | --- | --- | --- | --- | --- | --- | --- | --- | --- |
| control | 0.00 | 0.00 |  | control | 0.00 | 0.00 |  | control | 0.00 | 0.00 |
| Y: 0.1 mM pH7 | 46.27 | 1.86 |  | Y: 0.1 mM pH7.5 | 95.09 | 0.26 |  | Y: 0.1 mM pH8 | 87.58 | 0.46 |
| Pr: 0.1 mM pH7 | 75.89 | 1.10 |  | Pr: 0.1 mM pH7.5 | 63.61 | 1.64 |  | Pr: 0.1 mM pH8 | 94.03 | 0.33 |
| Nd: 0.1 mM pH7 | 86.57 | 0.37 |  | Nd: 0.1 mM pH7.5 | 90.47 | 0.10 |  | Nd: 0.1 mM pH8 | 98.64 | 0.08 |
| Gd: 0.1 mM pH7 | 81.15 | 0.16 |  | Gd: 0.1 mM pH7.5 | 79.37 | 0.72 |  | Gd: 0.1 mM pH8 | 97.11 | 0.05 |
| Tb: 0.1 mM pH7 | 77.22 | 0.93 |  | Tb: 0.1 mM pH7.5 | 67.41 | 0.03 |  | Tb: 0.1 mM pH8 | 99.98 | 0.00 |
| Dy: 0.1 mM pH7 | 72.90 | 1.54 |  | Dy: 0.1 mM pH7.5 | 76.97 | 0.18 |  | Dy: 0.1 mM pH8 | 76.97 | 0.18 |
| Y: 1 mM pH7 | 99.47 | 0.01 |  | Y: 1 mM pH7.5 | 92.43 | 0.27 |  | Y: 1 mM pH8 | 99.88 | 0.00 |
| Pr: 1 mM pH7 | 76.80 | 1.28 |  | Pr: 1 mM pH7.5 | 75.88 | 1.01 |  | Pr: 1 mM pH8 | 95.37 | 0.38 |
| Nd: 1 mM pH7 | 90.92 | 0.25 |  | Nd: 1 mM pH7.5 | 93.85 | 0.09 |  | Nd: 1 mM pH8 | 98.45 | 0.04 |
| Gd: 1 mM pH7 | 84.44 | 0.13 |  | Gd: 1 mM pH7.5 | 83.28 | 0.24 |  | Gd: 1 mM pH8 | 96.17 | 0.19 |
| Tb: 1 mM pH7 | 84.11 | 0.68 |  | Tb: 1 mM pH7.5 | 84.47 | 0.40 |  | Tb: 1 mM pH8 | 95.71 | 0.03 |
| Dy: 1 mM pH7 | 79.23 | 0.47 |  | Dy: 1 mM pH7.5 | 82.57 | 0.35 |  | Dy: 1 mM pH8 | 99.67 | 0.01 |

**Table S3: Adsorption capacity (qe) of Nd³⁺, Pr³⁺, Gd³⁺, Dy³⁺, Y³⁺, and Tb³⁺ on *Exiguobacterium* sp. SH31, at pH7 (A); pH 7.5(B); pH8 (C)**

| **(A)**  **Samples** | **qe (mg/g)** | **%RSD** |  | **(B)**  **Samples** | **qe (mg/g)** | **%RSD** |  | **(C)**  **Samples** | **qe (mg/g)** | **%RSD** |
| --- | --- | --- | --- | --- | --- | --- | --- | --- | --- | --- |
| Y: 0.1 mM pH7 | 1.723 | 5.10 |  | Y: 0.1 mM pH7.5 | 3.642 | 0.26 |  | Y: 0.1 mM pH8 | 3.354 | 0.46 |
| Pr: 0.1 mM pH7 | 3.662 | 0.16 |  | Pr: 0.1 mM pH7.5 | 3.583 | 0.72 |  | Pr: 0.1 mM pH8 | 4.383 | 0.05 |
| Nd: 0.1 mM pH7 | 3.795 | 0.37 |  | Nd: 0.1 mM pH7.5 | 3.966 | 0.10 |  | Nd: 0.1 mM pH8 | 4.324 | 0.08 |
| Gd: 0.1 mM pH7 | 3.662 | 0.16 |  | Gd: 0.1 mM pH7.5 | 3.583 | 0.72 |  | Gd: 0.1 mM pH8 | 4.383 | 0.05 |
| Tb: 0.1 mM pH7 | 3.359 | 0.93 |  | Tb: 0.1 mM pH7.5 | 2.933 | 0.03 |  | Tb: 0.1 mM pH8 | 2.933 | 0.03 |
| Dy: 0.1 mM pH7 | 2.540 | 1.54 |  | Dy: 0.1 mM pH7.5 | 2.682 | 0.18 |  | Dy: 0.1 mM pH8 | 2.682 | 0.18 |
| Y: 1 mM pH7 | 38.09 | 0.01 |  | Y: 1 mM pH7.5 | 35.40 | 0.27 |  | Y: 1 mM pH8 | 38.25 | 0.00 |
| Pr: 1 mM pH7 | 33.40 | 1.27 |  | Pr: 1 mM pH7.5 | 33.01 | 1.01 |  | Pr: 1 mM pH8 | 41.49 | 0.38 |
| Nd: 1 mM pH7 | 39.85 | 0.25 |  | Nd: 1 mM pH7.5 | 41.14 | 0.09 |  | Nd: 1 mM pH8 | 43.16 | 0.04 |
| Gd: 1 mM pH7 | 38.11 | 0.13 |  | Gd: 1 mM pH7.5 | 37.59 | 0.24 |  | Gd: 1 mM pH8 | 43.41 | 0.19 |
| Tb: 1 mM pH7 | 36.58 | 0.68 |  | Tb: 1 mM pH7.5 | 36.75 | 0.40 |  | Tb: 1 mM pH8 | 41.64 | 0.03 |
| Dy: 1 mM pH7 | 27.61 | 0.46 |  | Dy: 1 mM pH7.5 | 28.78 | 0.35 |  | Dy: 1 mM pH8 | 34.74 | 0.01 |

**Table S4: Separation factor RL calculated for Langmuir isotherm**

| **Samples** | **RL** |  | **Samples** | **RL** |  | **Samples** | **RL** |  | **Samples** | **RL** |
| --- | --- | --- | --- | --- | --- | --- | --- | --- | --- | --- |
| Y: 0.1 mM pH7 | 0.0755 |  | Nd: 0.1 mM pH7 | 0.2263 |  | Y: 1 mM pH7 | 0.4594 |  | Nd: 1 mM pH7 | 0.0415 |
| Y: 0.1 mM pH7.5 | 0.4777 |  | Nd: 0.1 mM pH7.5 | 0.2919 |  | Y: 1 mM pH7.5 | 0.0560 |  | Nd: 1 mM pH7.5 | 0.0600 |
| Y: 0.1 mM pH8 | 0.2657 |  | Nd: 0.1 mM pH8 | 0.7423 |  | Y: 1 mM pH8 | 0.7834 |  | Nd: 1 mM pH8 | 0.2023 |
| Pr: 0.1 mM pH7 | 0.1683 |  | Gd: 0.1 mM pH7 | 0.1683 |  | Pr: 1 mM pH7 | 0.0168 |  | Gd: 1 mM pH7 | 0.0239 |
| Pr: 0.1 mM pH7.5 | 0.1560 |  | Gd: 0.1 mM pH7.5 | 0.1560 |  | Pr: 1 mM pH7.5 | 0.0161 |  | Gd: 1 mM pH7.5 | 0.0223 |
| Pr: 0.1 mM pH8 | 0.5690 |  | Gd: 0.1 mM pH8 | 0.5690 |  | Pr: 1 mM pH8 | 0.0787 |  | Gd: 1 mM pH8 | 0.0905 |
| Tb: 0.1 mM pH7 | 0.1480 |  | Dy: 0.1 mM pH7 | 0.1541 |  | Tb: 1 mM pH7 | 0.0243 |  | Dy: 1 mM pH7 | 0.0232 |
| Tb: 0.1 mM pH7.5 | 0.1083 |  | Dy: 0.1 mM pH7.5 | 0.1766 |  | Tb: 1 mM pH7.5 | 0.0248 |  | Dy: 1 mM pH7.5 | 0.0276 |
| Tb: 0.1 mM pH8 | 0.1083 |  | Dy: 0.1 mM pH8 | 0.1766 |  | Tb: 1 mM pH8 | 0.0845 |  | Dy: 1 mM pH8 | 0.6002 |

**Table S5: EPS quantification by Congo red method of SH31 strain before and after metal exposure**

| **pH7** | **μg CR/OD600** | **Fold-changes** |  | **pH7.5** | **μg CR/OD600** | **Fold-changes** |  | **pH8** | **μg CR/OD600** | **Fold-changes** |
| --- | --- | --- | --- | --- | --- | --- | --- | --- | --- | --- |
| Control | 19.99 | 1.000 |  | Control | 45.54 | 1.000 |  | Control | 75.59 | 1.000 |
| 0.1 mM Nd | 19.82 | 0.9916 |  | 0.1 mM Nd | 40.44 | 0.8879 |  | 0.1 mM Nd | 62.10 | 0.8216 |
| 1 mM Nd | 19.58 | 0.9794 |  | 1 mM Nd | 46.34 | 1.018 |  | 1 mM Nd | 55.29 | 0.7315 |
| 0.1 mM Pr | 17.52 | 0.8763 |  | 0.1 mM Pr | 41.99 | 0.9219 |  | 0.1 mM Pr | 61.21 | 0.8098 |
| 1 mM Pr | 4.006 | 0.2004 |  | 1 mM Pr | 32.40 | 0.7113 |  | 1 mM Pr | 30.96 | 0.4096 |
| 0.1 mM Y | 18.15 | 0.9079 |  | 0.1 mM Y | 23.26 | 0.5107 |  | 0.1 mM Y | 20.36 | 0.2694 |
| 1 mM Y | 19.98 | 0.9993 |  | 1 mM Y | 28.62 | 0.6284 |  | 1 mM Y | 39.78 | 0.5263 |
| 0.1 mM Tb | 21.74 | 1.087 |  | 0.1 mM Tb | 38.43 | 0.8437 |  | 0.1 mM Tb | 47.66 | 0.6306 |
| 1 mM Tb | 5.802 | 0.2902 |  | 1 mM Tb | 37.53 | 0.8240 |  | 1 mM Tb | 38.90 | 0.5147 |
| 0.1 mM Gd | 23.69 | 1.185 |  | 0.1 mM Gd | 36.05 | 0.7916 |  | 0.1 mM Gd | 57.18 | 0.7565 |
| 1 mM Gd | 22.26 | 1.114 |  | 1 mM Gd | 28.39 | 0.6232 |  | 1 mM Gd | 45.85 | 0.6066 |
| 0.1 mM Dy | 22.20 | 1.110 |  | 0.1 mM Dy | 33.64 | 0.7387 |  | 0.1 mM Dy | 68.71 | 0.9090 |
| 1 mM Dy | 23.21 | 1.161 |  | 1 mM Dy | 31.17 | 0.6844 |  | 1 mM Dy | 62.22 | 0.8231 |

**Table S6: EPS quantification by Congo red method of SH31 strain before and after metal exposure in g/L**

| **Samples** | **Promedio**  **(μg CR/OD600)** | **OD_600_** | **g/L** |  | **Samples** | **Promedio**  **(μg CR/OD600)** | **OD_600_** | **g/L** |
| --- | --- | --- | --- | --- | --- | --- | --- | --- |
| pH7 -Control 1 | 0.9802 | 0.04085 | 4.085E-08 |  | pH7- 0,1mM Tb -1 | 1.0462 | 0.04350 | 4.350E-08 |
| pH7.5 -Control 1 | 1.2585 | 0.02290 | 2.290E-08 |  | pH7- 1mM Tb -1 | 1.0172 | 0.18655 | 1.866E-07 |
| pH8 -Control 1 | 1.1665 | 0.01280 | 1.280E-08 |  | pH7.5- 0,1mM Tb -1 | 1.2169 | 0.03245 | 3.245E-08 |
| pH7- 0,1mM Nd -1 | 1.0565 | 0.05330 | 5.330E-08 |  | pH7.5- 1mM Tb -1 | 1.2322 | 0.03165 | 3.165E-08 |
| pH7- 1mM Nd -1 | 1.0109 | 0.05163 | 5.163E-08 |  | pH8- 0,1mM Tb -1 | 1.1915 | 0.01880 | 1.880E-08 |
| pH7.5- 0,1mM Nd -1 | 1.2455 | 0.03080 | 3.080E-08 |  | pH8- 1mM Tb -1 | 1.1839 | 0.03950 | 3.950E-08 |
| pH7.5- 1mM Nd -1 | 1.2852 | 0.02050 | 2.050E-08 |  | pH7- 0,1mM Gd -1 | 1.1385 | 0.04807 | 4.807E-08 |
| pH8- 0,1mM Nd -1 | 1.2379 | 0.02320 | 2.320E-08 |  | pH7- 1mM Gd -1 | 1.0419 | 0.05020 | 5.020E-08 |
| pH8- 1mM Nd -1 | 1.2035 | 0.02760 | 2.760E-08 |  | pH7.5- 0,1mM Gd -1 | 1.2559 | 0.03620 | 3.620E-08 |
| pH7- 0,1mM Pr -1 | 0.8945 | 0.04110 | 4.110E-08 |  | pH7.5- 1mM Gd -1 | 1.2329 | 0.04160 | 4.160E-08 |
| pH7- 1mM Pr -1 | 0.8852 | 0.24905 | 2.491E-07 |  | pH8- 0,1mM Gd -1 | 1.0292 | 0.02175 | 2.175E-08 |
| pH7.5- 0,1mM Pr -1 | 1.2932 | 0.03080 | 3.080E-08 |  | pH8- 1mM Gd -1 | 0.9965 | 0.02815 | 2.815E-08 |
| pH7.5- 1mM Pr -1 | 1.2732 | 0.04070 | 4.070E-08 |  | pH7- 0,1mM Dy -1 | 1.1105 | 0.05003 | 5.003E-08 |
| pH8- 0,1mM Pr -1 | 1.2385 | 0.02590 | 2.590E-08 |  | pH7- 1mM Dy -1 | 1.0755 | 0.04633 | 4.633E-08 |
| pH8- 1mM Pr -1 | 1.1652 | 0.04295 | 4.295E-08 |  | pH7.5- 0,1mM Dy -1 | 1.2459 | 0.03500 | 3.500E-08 |
| pH7- 0,1mM Y -1 | 0.9952 | 0.05290 | 5.290E-08 |  | pH7.5- 1mM Dy -1 | 1.2415 | 0.03983 | 3.983E-08 |
| pH7- 1mM Y -1 | 0.9362 | 0.04495 | 4.495E-08 |  | pH8- 0,1mM Dy -1 | 0.9985 | 0.02035 | 2.035E-08 |
| pH7.5- 0,1mM Y -1 | 1.2189 | 0.05135 | 5.135E-08 |  | pH8- 1mM Dy -1 | 1.0162 | 0.01633 | 1.633E-08 |
| pH7.5- 1mM Y -1 | 1.2382 | 0.04165 | 4.165E-08 |  |  |  |  |  |
| pH8- 0,1mM Y -1 | 1.1165 | 0.05290 | 5.290E-08 |  |  |  |  |  |
| pH8- 1mM Y -1 | 1.1615 | 0.03305 | 3.305E-08 |  |  |  |  |  |

**SUPPLEMENTARY FIGURES**


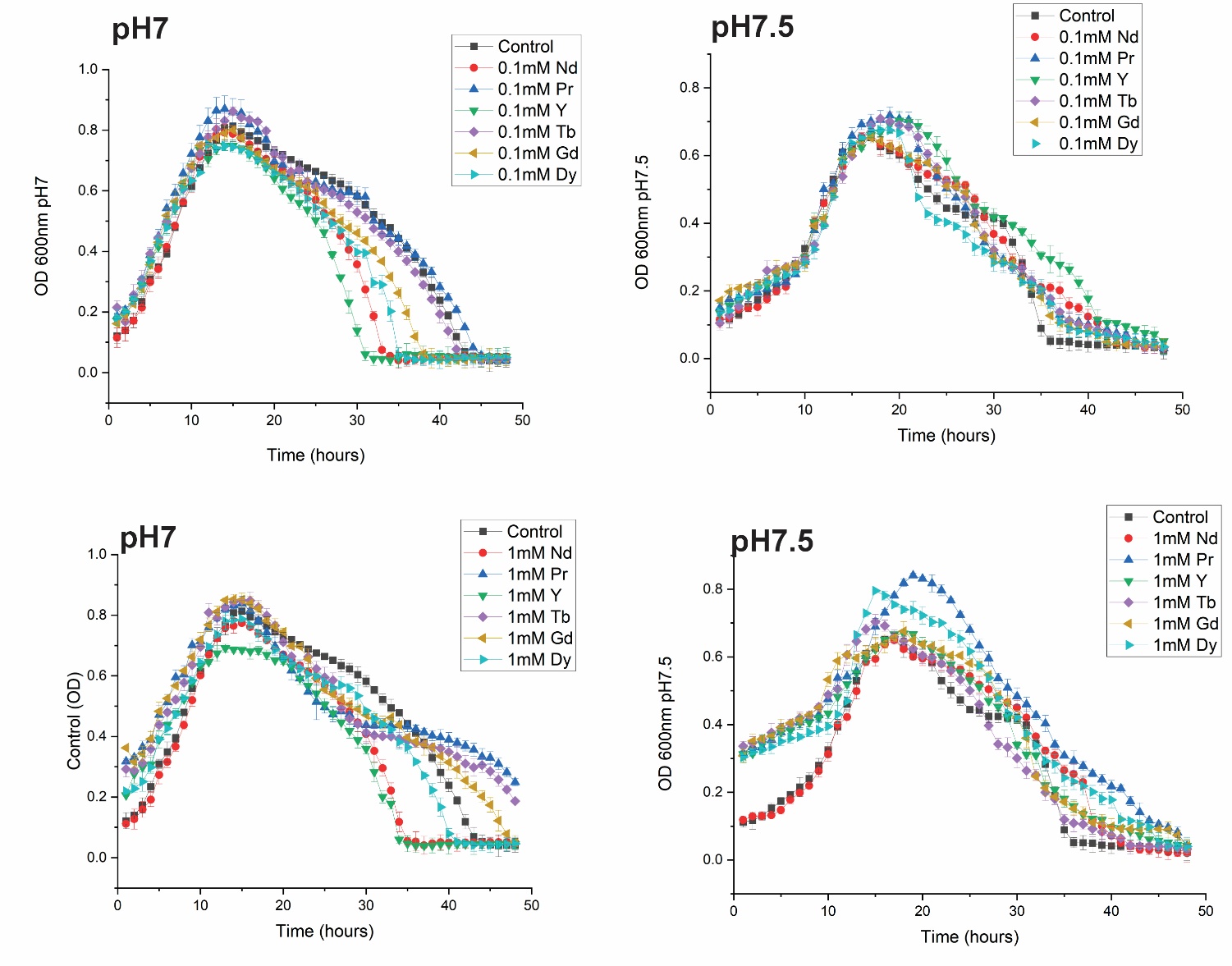


**Figure S1:** Growth curves of *Exiguobacterium* sp. SH31 at pH 7 and 7.5 and at 0.1mM and 1 mM of Nd, Pr, Y, Tb, Gd and Dy. Error bars represent three independent replicates.


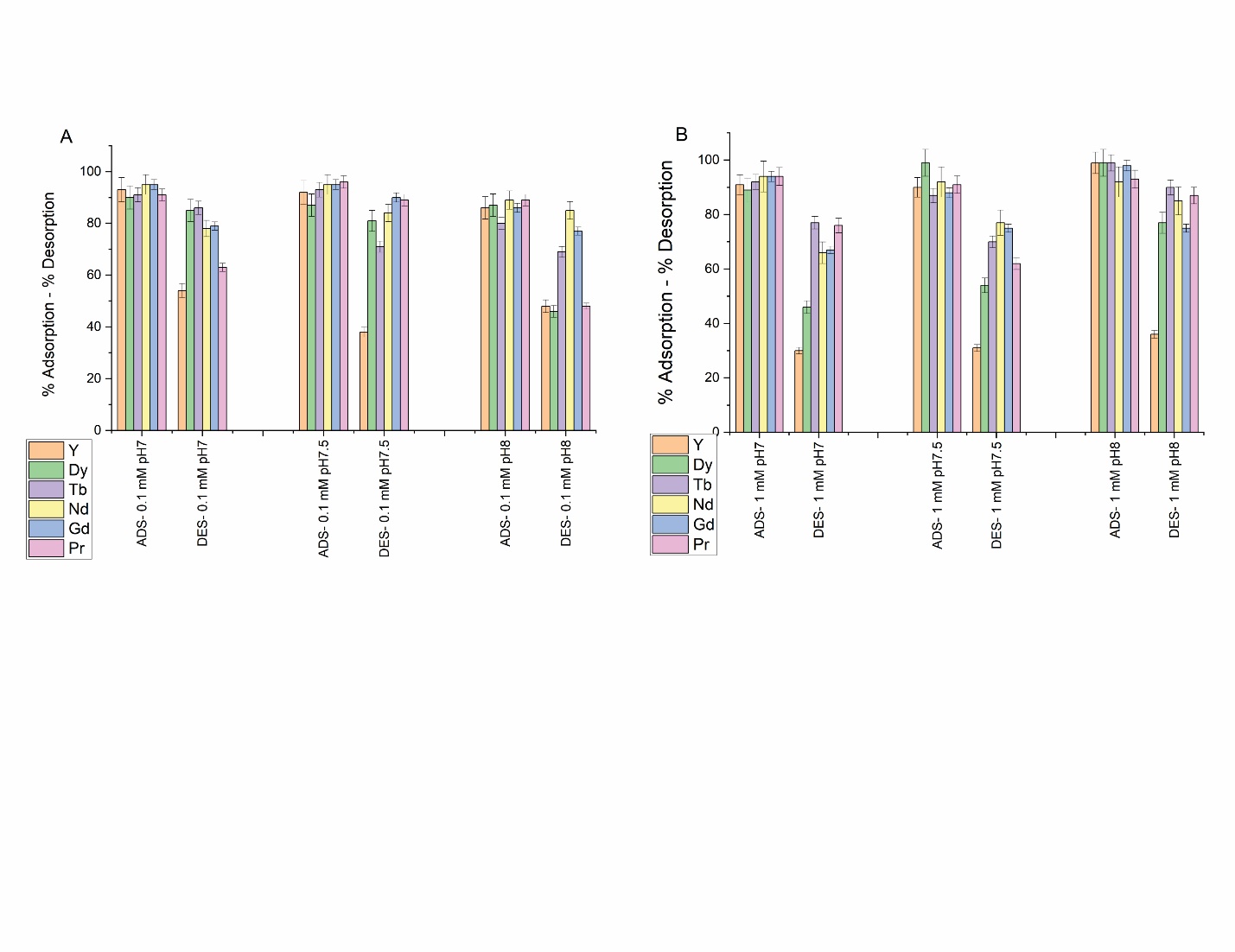


**Figure S2:** Metal adsorption and desorption of one cycle of use: a) Desorption of 0.1mM metal concentration bioadsorbed at pH 7, 7.5 and 8 and b) Desorption of 1mM metal concentration bioadsorbed at pH 7, 7.5 and 8.


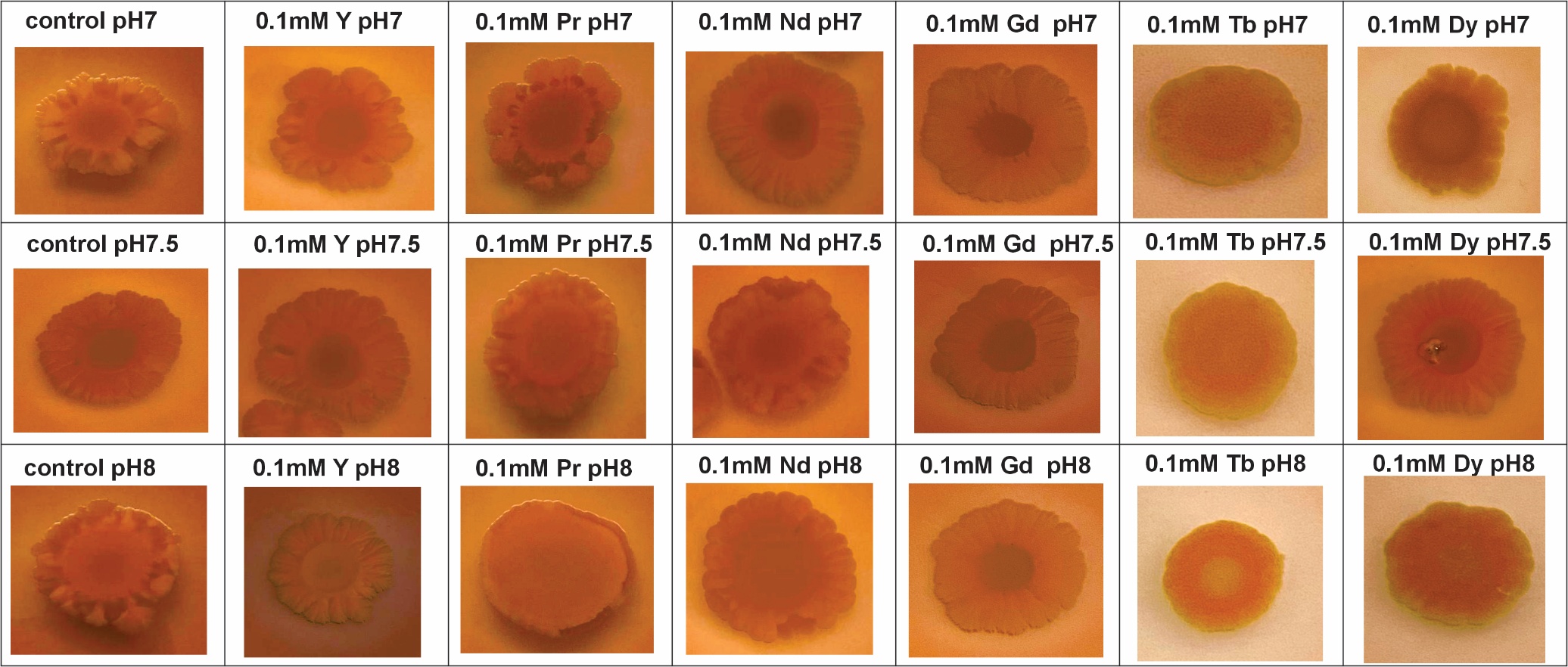


**Figure S3:** Congo Red plate assay under 0.1 mM REE conditions, showing qualitative EPS production across pH values. Color intensity and colony edge definition suggest variable EPS output in response to specific REEs.


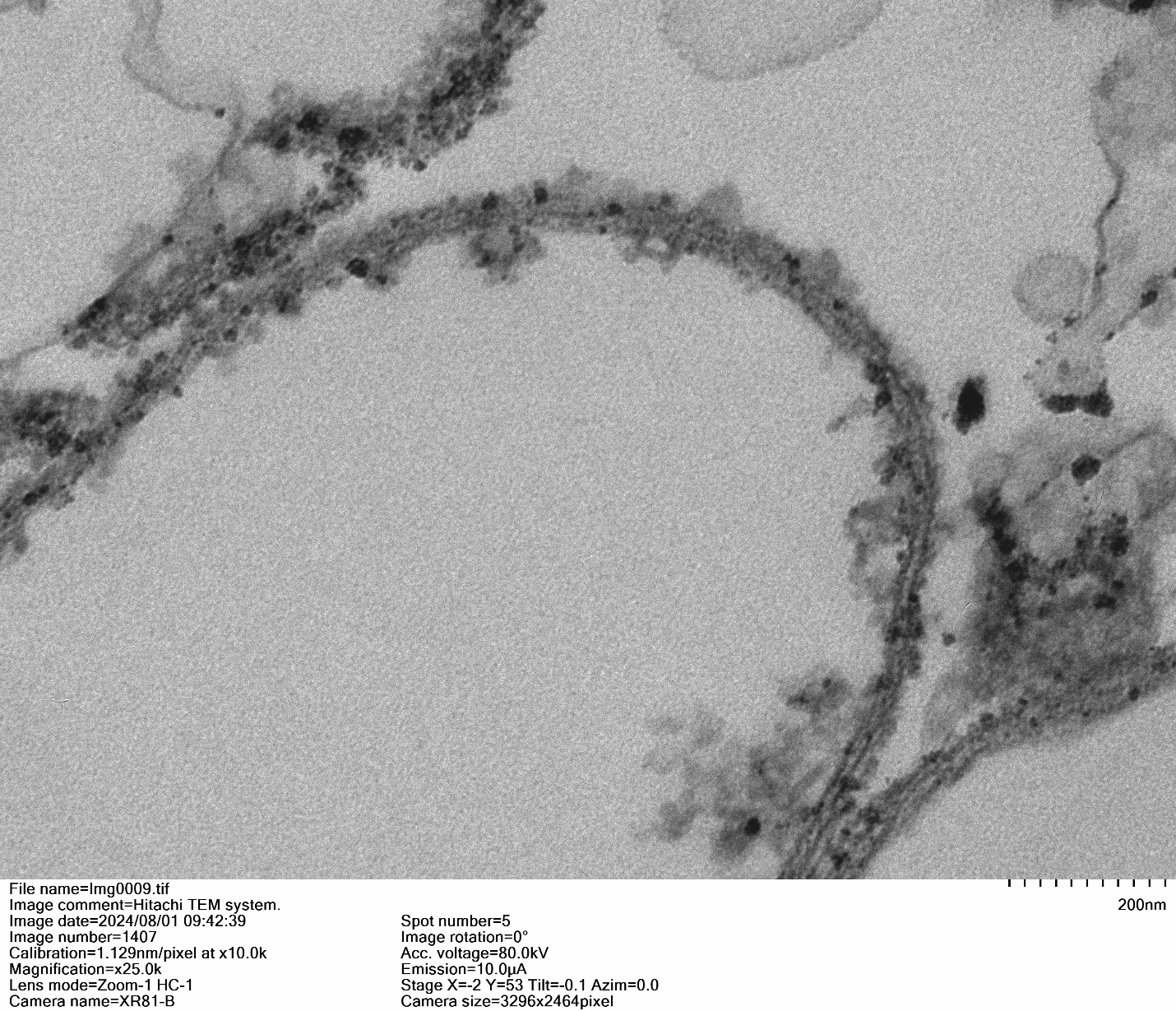


**Figure S5:** TEM image of SH31 strain exposed to Dy. The image shows the nano level of the agglomerated dense particles on the cell surface.


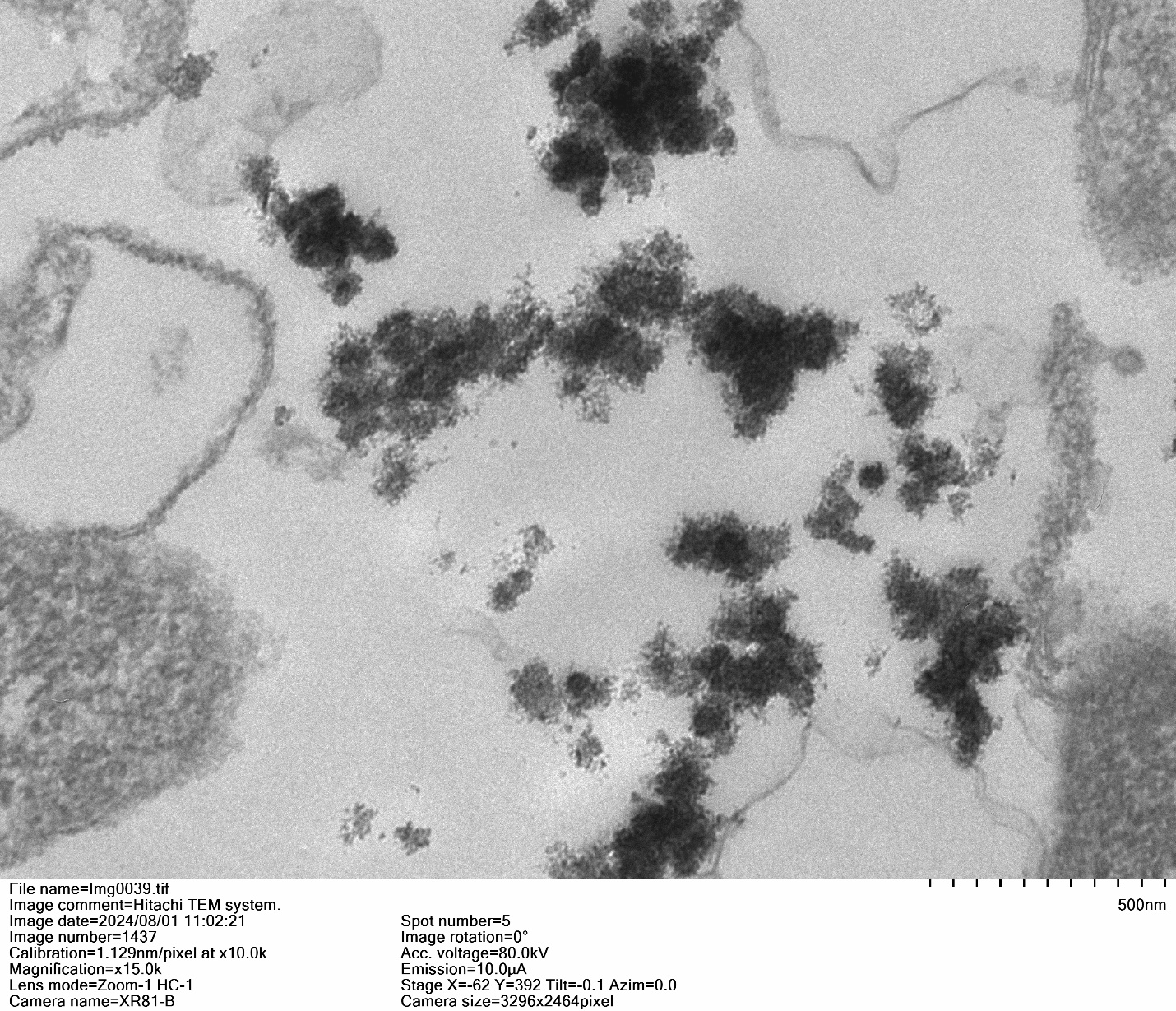


**Figure S6:** TEM image of SH31 strain exposed to Nd. The image shows the nano level of the agglomerated dense particles.
